# Influence of knee position and examiner-induced motion on the kinematics of the pivot shift

**DOI:** 10.1186/s40634-019-0183-7

**Published:** 2019-03-19

**Authors:** Jan-Hendrik Naendrup, Jason P. Zlotnicki, Conor I. Murphy, Neel K. Patel, Richard E. Debski, Volker Musahl

**Affiliations:** 1 Department of Trauma and Orthopaedic Surgery, Cologne Merheim Medical Center, Witten/Herdecke University, Cologne, Germany; 2 Orthopaedic Robotics Laboratory, Department of Bioengineering and Department of Orthopaedic Surgery, University of Pittsburgh, Center for Bioengineering - CNBIO, 300 Technology Drive, Pittsburgh, PA 15219 USA

**Keywords:** Knee, ACL, Quantitative pivot shift, Biomechanics, Education

## Abstract

**Background:**

Grading of the pivot shift test varies significantly depending on the examiner’s technique. Thus, the purpose of this study was to determine the influence of knee starting position and the magnitude of motion during the reduction event on the magnitude of the pivot shift test.

**Methods:**

Twenty-five clinical providers each performed a total of twenty pivot shift tests on one of two fresh-frozen cadaveric full lower extremity specimens with different grades of rotatory knee laxity. By means of ACL transection and lateral meniscectomy, one specimen was prepared to have a high-grade pivot shift and one to have a low-grade pivot shift. Six-degree-of-freedom kinematics were recorded during each pivot shift test using an electromagnetic-tracking-system. Successful pivot shift tests were defined and selected using an automated, mathematical algorithm based on the exceeding of a threshold value of anterior translation of the lateral knee compartment. The kinematics were correlated with the magnitude of anterior translation of the lateral knee compartment based on varying degrees of rotatory knee laxity using the Pearson correlation coefficient.

**Results:**

Only mild correlations between anterior translation of the lateral knee compartment and internal tibial rotation at the start of the reduction event were observed in both specimens. The ability to generate a successful reduction event was significantly dependent on the rotatory knee laxity, with a 54% success rate on the high-laxity specimen compared to a 30% success rate on the low-laxity specimen (*p* < 0.001). Nearly 80% of the variability of the anterior translation of the lateral knee compartment in both specimens was accounted for by external rotation during the reduction event (r = 0.847; *p* < 0.001). Varus rotation during the reduction event also showed a strong correlation with the anterior translation of the lateral knee compartment in the low-laxity specimen (r = 0.835; *p* < 0.001).

**Conclusion:**

Magnitude of motion during the reduction event affected the magnitude of anterior translation of the lateral knee compartment more than the starting position. External rotation during the reduction event accounted for most of the variability in the pivot shift test. More uniform maneuvers and improved teaching are essential to generate repeatable quantitative results of the pivot shift test.

## Background

Despite variation in the motion used during the pivot shift test, the majority of techniques involve flexion of the knee while the tibia is internally rotated and a gentle valgus stress is applied (Hoshino et al. 2012c; Kuroda et al. 2012b; Noyes et al. 1991; Musahl et al. 2012). In cases of anterior cruciate ligament (ACL) deficiency, the lateral tibial plateau translates anteriorly from its physiological position beneath the femoral condyle and this tibial subluxation is followed by a posterior translation of the tibia back to its physiologic position, called the reduction event. The reduction event usually occurs when the knee reaches 25 to 40 degrees of flexion (Galway and MacIntosh 1980; Losee 1983) In order for the reduction event to occur, the examiner needs to release the applied internal torque and valgus stress as the passive pulling of the iliotibial tract initiates the posteriorly directed translation of the lateral tibial plateau. Due to the multi-planar motions, the validity of the pivot shift test depends on the experience, technique, and technical skills of the examiner and as a result the inter-examiner reliability is low. (Noyes et al. 1991; Musahl et al. 2012; Peeler et al. 2010)

In addition to a lack of uniformity with respect to the technique, there is no generally accepted method for teaching a repeatable pivot shift test. While some clinicians learn the pivot shift test through hands-on instructions in the clinic or operating room, others learn the test passively through instructional videos or textbooks. Non-standardized teaching methods for the pivot shift test may contribute to variability of techniques among orthopaedic surgeons and may limit comparability of the results (Kuroda et al. 2012b). For teaching purposes, knowledge of the examiner-induced motions due to appropriate loads applied may be essential to generate a successful pivot shift test.

Given the development of new methodologies that allow for the objective quantification of the pivot shift test (Bedi et al. 2010; Hoshino et al. 2007; Kuroda and Hoshino 2016; Kuroda et al. 2012a; Lopomo et al. 2013; Lopomo et al. 2012; Muller et al. 2015; Muller et al. 2016; Müller 1931; Nakamura et al. 2017; Zaffagnini et al. 2016), a uniform and repeatable technique that produces comparable and reproducible quantitative results is needed to enable longitudinal monitoring of knee laxity during the rehabilitation process. In order to improve the execution of the pivot shift test, the required position of the knee at the start of the reduction event and examiner-induced motions needed during the reduction event to generate a successful pivot shift test must be better understood. Therefore, the purpose of this study was to determine the influence of starting knee position and examiner-induced motion during the reduction event on the anterior translation of the lateral knee compartment during the pivot shift test. It was hypothesized that the knee starting position and examiner-induced motion during the reduction event along the varus/valgus and internal/external rotation axes would correlate significantly with the anterior translation of the lateral knee compartment.

## Methods

Approval for this study was obtained from the local IRB and the Committee for Oversight in Research Involving Decedents (CORID) in accordance with the ethical standards of the Declaration of Helsinki (1964) and its later amendments. Two cadaveric fresh-frozen full lower extremity specimens were thawed for 36 h at room temperature before the start of the experimental protocol. Prior to testing, each specimen was manually, radiographically, and arthroscopically examined by a senior orthopaedic surgeon in order to ensure that there were no pre-existing osteoarthritis or injuries to the ACL, posterior cruciate ligament, menisci, or cartilage. Subsequently, the ACL was transected and removed arthroscopically and a lateral meniscectomy was performed to generate rotatory laxity in each knee. Specimen 1 had a high-grade pivot shift test (“gross clunk”) and specimen 2 had a gliding pivot shift test based on a manual exam by the senior orthopaedic surgeon using the standard subjective International Knee Documentation Committee (IKDC) 2000 form of “glide”, “clunk”, or “gross clunk”. (Hefti et al. 1993; Irrgang et al. 1998)

Twenty-five subjects, representing a wide range of levels of clinical expertise and experience, were recruited to participate in this study. Informed consent was obtained from all participants prior to the start of the study. The study population consisted of five subjects in each of the five subgroups: sports medicine fellows, orthopaedic residents, physical therapists, athletic trainers, and medical students. Each subject performed 20 pivot shift tests on one of the two full lower extremity specimens while the six degree-of-freedom (DOF) kinematics were recorded. The pivot shift tests were performed using recently proposed maneuvers, consisting of internal rotation, valgus stress and continuous knee flexion that allows for reduction of the lateral tibial plateau (Musahl et al. 2012). An instructional video of the technique was provided during testing. Additionally, an experienced senior orthopaedic surgeon performed 10 pivot shift tests on both specimens and the kinematics were recorded for use as a gold standard reference.

**Fig. 1:**
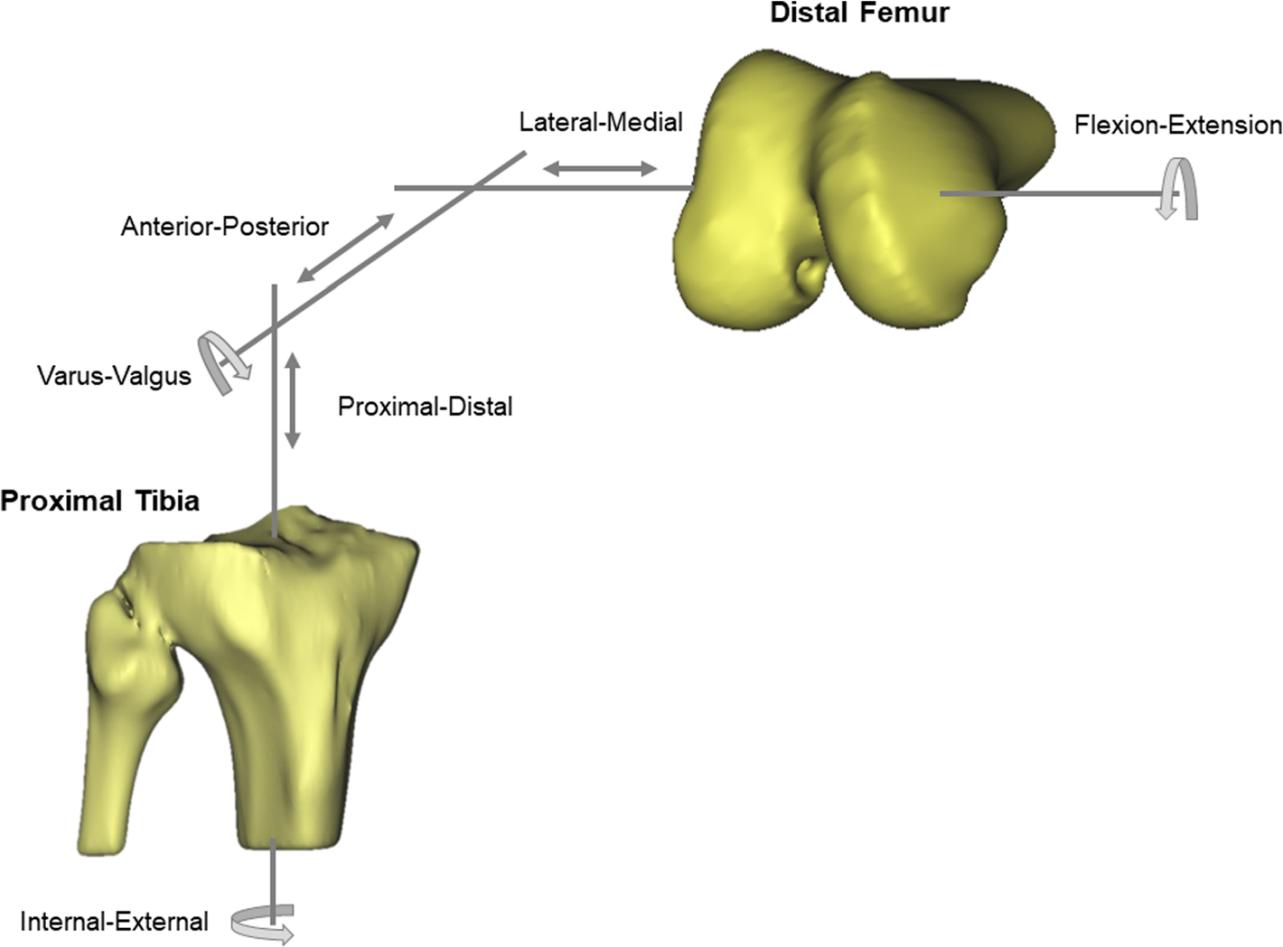
Grood/Suntay joint coordinate system, demonstrated on a bony knee model. Medial-lateral translation and flexion-extension occurs along/about the femoral fixed axis, positioned through both femoral epicondyles and consequently perpendicular to the sagittal plane. Proximal-distal translation and internal-external rotation occurs along/ about the axis, fixed to the tibial shaft. Anterior-posterior translation and valgus-varus rotation occurs along/about the floating axis, defined by the cross product of the femoral and tibial axes and therefore perpendicular to both body fixed axes

**Fig. 2:**
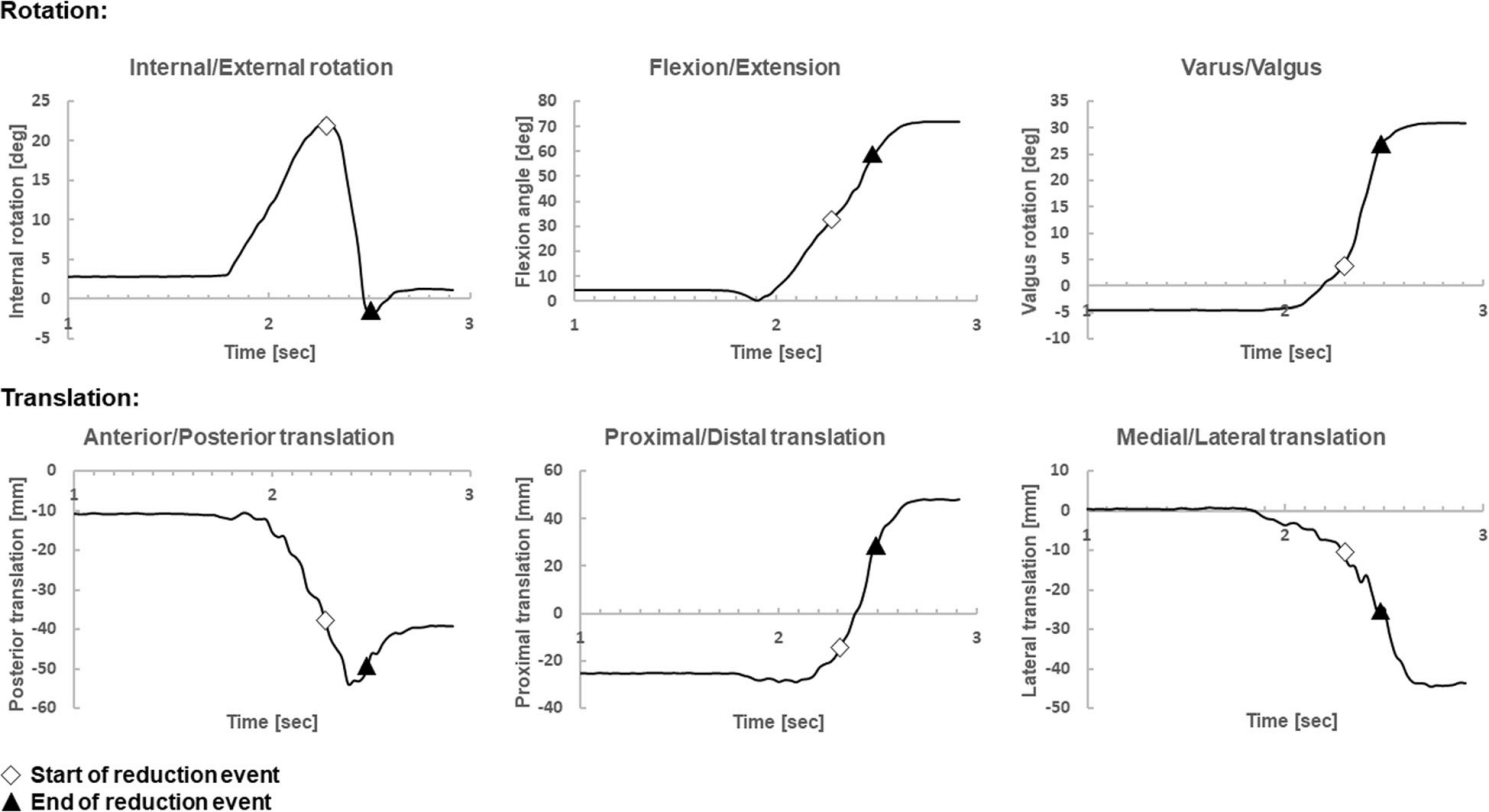
A typical set of six degree of freedom kinematics recorded while the pivot shift test is performed by an experienced senior orthopaedic surgeon. The top row shows rotational DOF as a function of time. The bottom row shows translational DOF as a function of time. The start of the reduction event is labelled with a white rhombus, the end of the reduction event is labelled with a black triangle

Six DOF kinematics were recorded using an electromagnetic tracking system (Nest of Birds, Ascension Technology, Shelburne, VT) with an accuracy of 0.5 mm for translation and 0.5° for rotation. Electromagnetic sensors were affixed to the femur and the tibia approximately 20 cm proximally and distally from the joint, respectively. A third sensor, attached to a stylus, was used to digitize the medial/lateral femoral epicondyle, medial/lateral tibial plateau, fibular head, Gerdy’s tubercle, and two landmarks along the shaft of tibia and femur. With this information, the Grood/Suntay joint coordinate system (see Fig. 1) was determined to describe the relative position and orientation of the tibia with respect to the femur (Grood and Suntay 1983). In addition, the anterior translation of the lateral knee compartment during the pivot shift test was calculated, representing an objective measure that quantifies the rotatory knee laxity of the pivot shift test (Hoshino et al. 2012b; Musahl et al. 2012). A typical set of 6 DOF kinematics for the performance of one pivot shift test by an experienced senior orthopaedic surgeon is shown in Fig. 2.

**Fig. 3:**
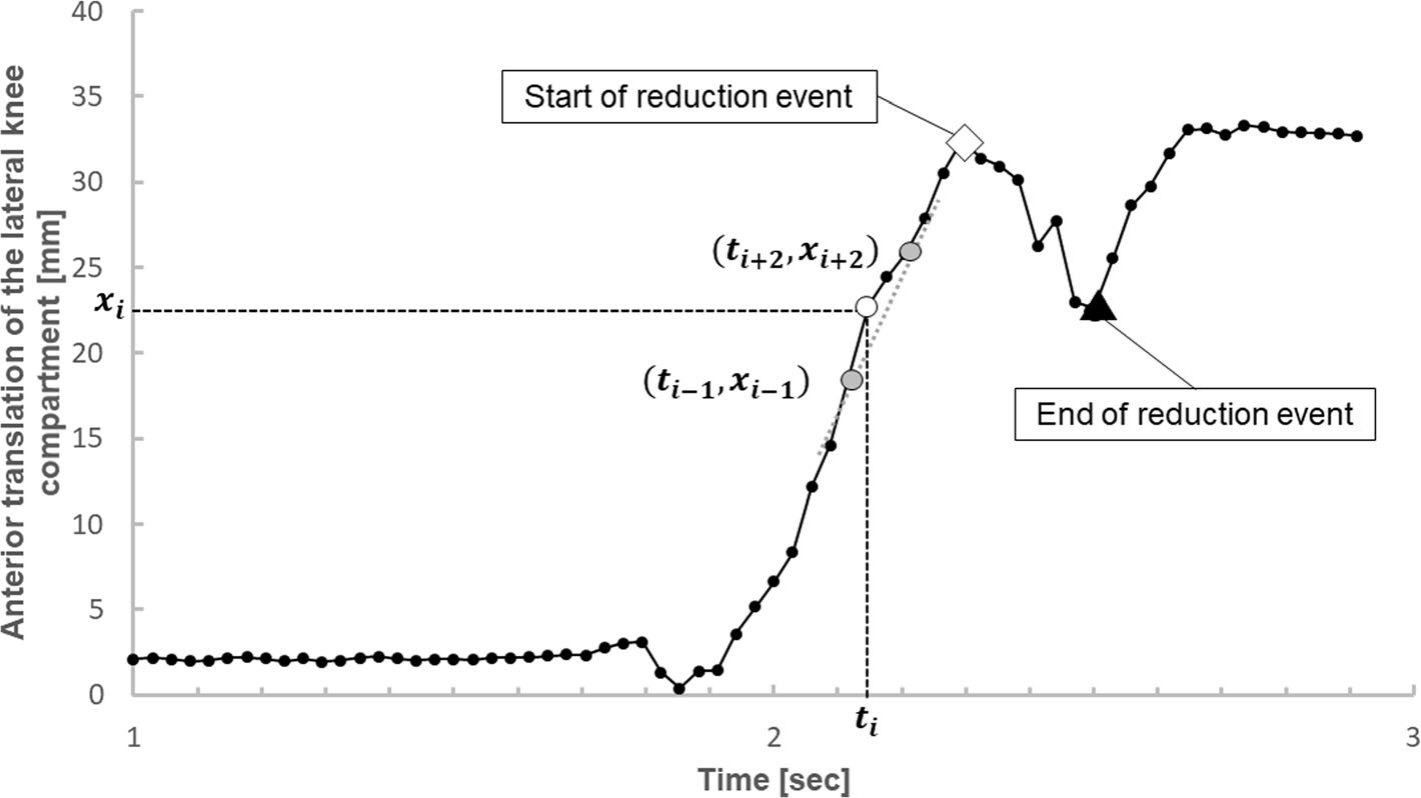
Illustration of the running slope of four values (dashed grey line) for the measured value (*t*_*i*_, *x*_*i*_) (white mark), calculated based on the values (*t*_*i* − 1_, *x*_*i* − 1_) and (*t*_*i* + 2_, *x*_*i* + 2_) (grey marks). Furthermore, the start of the reduction event is labelled with a white rhombus, the end of the reduction event is labelled with a black triangle. The difference in anterior translation of the lateral knee compartment between the start and the end of the reduction event is used for the quantification of the pivot shift test

Quantitative criteria were utilized to determine which pivot shift tests were successful. An extended slope ${s}_i=\frac{x_{i+2}-{x}_{i-1}}{t_{i+2}-{t}_{i-1}}$ was calculated to smooth short-term fluctuations in the slope of the anterior translation of the lateral knee compartment as a function of time (see Fig. 3). A change in sign of the extended running slope was used to select the maximum anterior translation of the lateral knee compartment (*s*_*max*_ = 0, change from positive to negative slope), which marks the maximal subluxation of the tibia and the start of the reduction event; and the minimum anterior translation of the lateral knee compartment (*s*_*min*_ = 0, change from negative to positive slope, which marks the end of the reduction event (see Fig. 3)). Trials without a change in sign of the slope were graded as a negative pivot shift test. The difference between maximum and minimum anterior translation of the lateral knee compartment was calculated and averaged for the trials that had a change in sign of the slope. In addition, pivot shift tests were considered successful only if this difference was greater than the square root of the standard deviation of all anterior translation of the lateral knee compartment values. The starting position of the reduction event was defined as the 6 DOF position of the knee at the time of maximum anterior translation of the lateral knee compartment (Fig. 3). Examiner-induced motion during the reduction event was defined as the difference between the 6 DOF positions at the maximum and minimum anterior translation of the lateral knee compartment. Both starting position and examiner-induced motion of positive pivot shift tests were used for the statistical analysis.

Using SPSS (version 20.0, SPSS Inc., Chicago, USA), two-tailed t-tests were used to determine differences between the high-laxity specimen (specimen 1) and the low-laxity specimen (specimen 2) regarding the knee position at the start of the reduction event and the amount of motion during the pivot shift test. Pearson-correlations were calculated to determine the relationship between the knee position at the start of the reduction event and the motion during the pivot shift test as well as the anterior translation of the lateral knee compartment. Correlation coefficients were defined as “weak positive correlation” (0.20–0.39), “moderate positive correlation” (0.40–0.59), “strong positive correlation” (0.60–0.79), and “very strong positive correlation” (0.80–1.00). Multiple factor analysis of variance (ANOVA) were applied to determine what amount of variability of the anterior translation of the lateral knee compartment is accounted for by of each DOF of motion. Statistical significance was set at *p* < 0.05.

## Results

A total of 500 pivot shift tests performed by the five groups of clinicians and 20 pivot shift tests performed by the senior orthopaedic surgeon (gold standard) were recorded on both specimens (190 on the high-laxity specimen, 330 on the low-laxity specimen). Of the 520 pivot shift tests, 319 (61.3%) were excluded from the analysis since they did not meet the criteria of a successful pivot shift test. Of the 201 positive pivot shift tests performed, 103 positive pivot shift tests were performed on the high-laxity specimen and 98 on the low-laxity specimen. The reduction event of the pivot shift test occurred in an average time interval of 0.18 s.

Significant differences in the position of the tibia relative to the femur at the start of the reduction event between the two specimens were found, especially with respect to internal rotation and lateral translation. The starting position of the reduction event in the high-laxity specimen was 17.3° greater in internal rotation than the low-laxity specimen (*p* < 0.001) and was 5.5 mm greater in lateral translation than the low-laxity specimen (*p* < 0.01) (see Table 1). Despite the different laxity patterns, no differences were detected with respect to the flexion angle at the start of the reduction event with 34.9 ± 9.1° in the high-laxity specimen and 34.7 ± 7.8° in the low-laxity specimen. Differences in motion during the reduction event with varying rotatory knee laxity were also found (see Table 1). In the high-laxity specimen, the external rotation, lateral translation, and the proximal translation were twice as high as those for the low-laxity specimen (*p* < 0.001). The anterior translation of the lateral knee compartment was doubled in the high-laxity specimen, 17.3 ± 8.8 mm, compared to the low-laxity specimen, 8.8 ± 10.5 mm.

**Table 1:** Knee position at the start of the reduction event of the pivot shift test and examiner-induced motion during the reduction event (calculated as difference Δ between joint position at the start and at the end of the reduction event of the pivot shift) in the high grade laxity specimen and the low grade laxity specimen based on the Grood/Suntay coordinate system. Values are represented as average ± standard deviation

DOF	Specimen	Starting position	Examiner-induced motion
Internal/External rotation (°) (+ = internal rotation, − = external rotation)	High grade laxity	28.7 ± 15.5 *	− 19.3 ± 7.9*
	Low grade laxity	11.4 ± 5.5 *	− 10.0 ± 9.2*
Flexion (°)	High grade laxity	34.9 ± 9.1	–
	Low grade laxity	34.7 ± 7.8	–
Varus/Valgus rotation (°) (+ = varus, − = valgus)	High grade laxity	−4.8 ± 8.7	12.9 ± 6.8 *
	Low grade laxity	−2.3 ± 22.2	10.3 ± 6.7 *
Anterior/Posterior translation (mm) (+ = anterior, − = posterior)	High grade laxity	−30.9 ± 14.7	−16.1 ± 10.5
	Low grade laxity	− 30.4 ± 15.9	−14.3 ± 35.9
Distal/Proximal translation (mm) (+ = distal, − = proximal)	High grade laxity	−23.1 ± 17.5	− 44.8 ± 13.6 *
	Low grade laxity	−21.9 ± 19.8	− 24.5 ± 10.9 *
Medial/Lateral translation (mm) (+ = medial, − = lateral)	High grade laxity	−11.2 ± 14.9 *	− 12.6 ± 6.1 *
	Low grade laxity	−5.7 ± 5.1 *	− 5.4 ± 6.6 *
Anterior translation of lateral knee compartment (mm)	High grade laxity	49.9 ± 13.5	17.3 ± 8.8 *
	Low grade laxity	50.9 ± 8.6	8.8 ± 10.5 *

* *p* < 0.05

Weak and moderate positive correlations (r = 0.43 in high-laxity specimen, r = 0.35 in low-laxity specimen, *p* < 0.05) between the anterior translation of the lateral knee compartment and the internal rotation of the tibia at the start of the reduction event were found in both specimens (Table 2). The flexion angle at which the reduction event started illustrated strong and very strong positive correlations with the posterior translation (r = 0.86 in high-laxity specimen, r = 0.67 in low-laxity specimen, *p* < 0.05) and the proximal translation (r = 0.83 in high-laxity specimen, r = 0.56 in low-laxity specimen, p < 0.05) positions at the start of the reduction event (Table 2). The amount of valgus rotation at the start of the reduction event showed a strong positive correlation with the flexion angle at the start of the reduction event only in the high-laxity specimen (r = 0.73, *p* < 0.001).

**Table 2:** Correlation of the knee position at the start of the reduction event of the pivot shift test with (A) the ensuing anterior translation of the lateral knee compartment and with (B) the flexion angle at the start of the reduction event in the high grade laxity specimen and low grade laxity specimen

DOF	Specimen	Pearson coefficient: Anterior translation of lateral knee compartment	Pearson coefficient: Flexion angle at start of reduction event
Internal/External rotation (+ = internal rotation, − = external rotation)	High grade laxity	0.433*	− 0.193
	Low grade laxity	0.346*	0.272*
Varus/Valgus rotation (+ = varus, − = valgus)	High grade laxity	−0.149	0.730*
	Low grade laxity	−0.059	0.217*
Anterior/Posterior translation (+ = anterior, − = posterior)	High grade laxity	0.357*	− 0.856*
	Low grade laxity	−0.087	− 0.669*
Distal/Proximal translation (+ = distal, − = proximal)	High grade laxity	−0.154	0.827*
	Low grade laxity	0.343*	0.559*
Medial/ Lateral translation (+ = medial, − = lateral)	High grade laxity	−0.217*	− 0.259*
	Low grade laxity	−0.290*	− 0.437*
Anterior translation of lateral knee compartment	High grade laxity	−0.010	0.552*
	Low grade laxity	0.296*	0.725*

* *p* < 0.05

Strong and very strong positive correlations (r = 0.78 in high-laxity specimen, r = 0.84 in low-laxity specimen, *p* < 0.05) were obtained between the anterior translation of the lateral knee compartment and the motion of the knee (Table 3), especially for the external rotation during the reduction event. Multiple factors ANOVA showed that external rotation accounts for 78.8% of the variability of the anterior translation of the lateral knee compartment in both specimens. Additionally, varus rotation during the reduction event showed strong correlations, but only in the low-laxity specimen (*p* < 0.001).

**Table 3:** Correlation between examiner-induced motion during the reduction event of the pivot shift test and the anterior translation of the lateral knee compartment in the high grade laxity specimen and the low grade laxity specimen

DOF	Pivot shift	Pearson coefficient: Anterior translation of lateral knee compartment
Internal/External rotation (+ = internal rotation, − = external rotation)	High grade laxity	−0.776*
	Low grade laxity	−0.843*
Varus/Valgus rotation (+ = varus, − = valgus)	High grade laxity	−0.017
	Low grade laxity	0.835*
Anterior/Posterior translation (+ = anterior, − = posterior)	High grade laxity	−0.126
	Low grade laxity	−0.397*
Distal/Proximal translation (+ = distal, − = proximal)	High grade laxity	−0.445*
	Low grade laxity	−0.363*
Medial/Lateral translation (+ = medial, − = lateral)	High grade laxity	−0.227*
	Low grade laxity	−0.307*

* *p* < 0.05

## Discussion

This study showed that the quantitative measures of the pivot shift test depend on the knee position at the start of the reduction event and the motion of the knee during the reduction event along the internal/external and varus/valgus DOFs. Additionally, the importance of the varus/valgus rotation varied depending on the rotatory knee laxity pattern of the specimen. Regardless of the rotatory knee laxity pattern, the motion during the reduction event showed stronger correlations with anterior translation of the lateral knee compartment than the position at the start of the reduction event. External rotation during the reduction event constituted the most important kinematic component for the quantitative measure of the pivot shift test, accounting for 78.8% of the variability of anterior translation of the lateral knee compartment. However, variability in the performance of the pivot shift test persisted throughout the experiment as reflected by the overall success rate of only 40% and the high standard deviations in the kinematic data (Table 1).

The measured values for the external rotation are in agreement with data from previously published literature. Bull et al. intra-operatively measured the external rotation of the knee of 10 patients with an ACL-deficiency before reconstruction and found an external rotation of 13° and a standard deviation of 8° (Bull et al. 2002). This external rotation lies between the average measurements of the high-laxity and low-laxity specimen in this study and the standard deviations are also comparable. A cadaveric study found similar amounts of external rotation during the reduction event (17 ± 11°) for a simulated pivot shift test with loading of the iliotibial tract and application of a valgus stress by hanging weights on an intramedullary tibial rod (Bull et al. 1999). The current study showed that external rotation during the reduction event constituted the most important kinematic component for the quantitative measure of the pivot shift test and was more important than the internal rotation at the start of the reduction event.

In order for a positive pivot shift test to occur, the examiner needs to release the valgus stress, applied prior to the start of the reduction event, and allow for a varus rotation during the reduction event. In contrast to the high-laxity specimen, the amount of varus rotation during the reduction event strongly correlated with the magnitude of anterior translation of the lateral knee compartment in the low-laxity specimen. The influence of the amount of valgus stress prior to the reduction event on the anterior translation of the lateral knee compartment during a simulated pivot shift test in cadaveric specimens was recently examined. There is a threshold of valgus stress after which greater valgus stress did not lead to increased anterior translation of the lateral knee compartment (Citak et al. 2012). However, based on the results of this study a greater valgus stress should lead to greater valgus rotation at the start of the reduction event and consequently increase the anterior translation of the lateral knee compartment. Therefore, new teaching methods for the pivot shift test need to emphasize better control of the applied valgus stress and future studies may require analysis of the applied loads.

Several previous studies have analyzed the flexion angle at the start of the reduction event of the pivot shift test and reported varying results in the range of 25° to more than 40° (Bull et al. 1999; Bull et al. 2002; Losee 1983; Slocum et al. 1976). Intraoperative measurements revealed a flexion angle at the start of the reduction event of 36 ± 9°, closely corresponding with our data (Bull et al. 2002). Interestingly, no differences of the flexion angles at the start of the reduction event were found between the high and low-laxity specimen. In both rotatory knee laxity patterns, good correlations exist with the posterior and proximal translation of the lateral knee compartment and the flexion angle at the start of the reduction event. A strong positive correlation also existed with the valgus rotation position only in high laxity specimen. The relatively high standard deviations reported in the literature and also present in this study could be attributed to the non-uniformity of the motions for each DOF during the pivot shift test. The dependence of the flexion angle at the start of the reduction event on kinematics in different DOF is similar to previous observations that the flexion angle at the start of the reduction event varies depending on the “balance between applied valgus and iliotibial tract loads”(Bull et al. 1999).

Standardizing the hand position and motions of the pivot shift test were shown to improve the consistency of the quantitative evaluation of rotatory knee laxity in a group of experienced senior orthopaedic surgeons (Hoshino et al. 2012a). Therefore, a standardized technique for this clinical exam seems to be an essential factor in minimizing variability between examiners and obtaining reliable results, as different techniques of the pivot shift test result in relevant biomechanical differences.(Kitamura et al. 2013) Based on the findings of this study, the magnitude of motion during the reduction event is more important for the quantitative measure of the pivot shift test than the knee position at the start of the reduction event. Therefore, teaching of the pivot shift test should focus on the motion during the reduction event, especially the external rotation. However, the reduction event of the pivot shift test occurred in a time interval of 0.18 s. Therefore, there is a small window of time for the examiner to allow the ideal reduction by releasing the applied stress or inducing motions to accentuate the details of rotatory knee laxity. This finding might constitute a possible explanation for high inter- and even intra-examiner variability of the pivot shift test. Currently, there is no supplementary equipment or simulation to facilitate the learning of clinical knee exams like the pivot shift test. Future research will focus on the development of new learning methods to enable unexperienced clinicians to induce the correct motions during the reduction event, as it is questionable if passive teaching or occasional hands-on teaching is sufficient to teach the needed muscle memory (Brashers-Krug et al. 1996) (Krakauer and Shadmehr 2006) to perform a repeatable pivot shift..

This study has several limitations. Beside the limited number of specimens, one limitation of this study is that loads applied by the examiner during the pivot shift could not be assessed. Analysis of the loads would be especially important with regards to the valgus torque applied at the start of the reduction event, since more valgus rotation correlated with increased anterior translation of the lateral knee compartment. In addition, the grading of the pivot shift test is influenced by the hip position, possibly due to tensioning of the iliotibial band (Bach et al. 1988). However, the hip position was not analyzed during this study. The results of the present study are based on biomechanical testing on cadaveric specimens without muscle activation and guarding, so care must be taken when transferring results into clinical settings. Also, the precision of electromagnetic tracker systems is limited with an accuracy of 0.5 mm for translation and 0.5° for rotation.

## Conclusion

Based on the findings of this study, a controlled position at the start of the reduction event and examiner-induced motion during the reduction event of the pivot shift test along the internal/external and varus/valgus rotation axes are required to achieve repeatable quantitative measurements of the anterior translation of the lateral knee compartment. The examiner-induced motion during the reduction event showed stronger correlations with the measurements of the anterior translation of the lateral knee compartment than the position at the start of the reduction event did. In order to achieve repeatable and comparable quantitative results of the pivot shift test, more uniform motions should to be implemented and the development of new teaching methods could aid in decreasing variability of the pivot shift test. Ultimately, this would lead to improved use of the pivot shift test in the assessment of rotatory knee laxity in patients with ACL injury throughout their recovery.

## Abbreviations

ACL: Anterior cruciate ligament
ANOVA: Analysis of variance
DOF: Degree of freedom
